# Effects of Interoceptive Sensibility on Mental Health during the Coronavirus Disease 2019 Pandemic

**DOI:** 10.3390/ijerph18094616

**Published:** 2021-04-27

**Authors:** Naho Suzuki, Tetsuya Yamamoto, Chigusa Uchiumi, Nagisa Sugaya

**Affiliations:** 1 Graduate School of Sciences and Technology for Innovation, Tokushima University, Tokushima 770-8502, Japan; gypll.82@gmail.com; 2 Graduate School of Technology, Industrial and Social Sciences, Tokushima University, Tokushima 770-8502, Japan; uchiumi@tokushima-u.ac.jp; 3 Unit of Public Health and Preventive Medicine, School of Medicine, Yokohama City University, Yokohama 236-0004, Japan; nagisa_s@yokohama-cu.ac.jp

**Keywords:** interoception, interoceptive sensibility, body awareness, COVID-19, quarantine, mental health, depression, anxiety, somatization symptoms, multidimensional assessment of interoceptive awareness

## Abstract

The current coronavirus disease 2019 pandemic has been reported to influence interoceptive sensibility. This study focused on adaptive and maladaptive aspects of interoceptive sensibility and examined how each aspect of interoceptive sensibility affects depression, anxiety, and somatization symptoms under the mild lockdown in Japan, which was not enforceable and a non-punitive lockdown. We used data from 10,672 participants who lived in prefectures where the emergency declaration was first applied in Japan. Interoceptive sensibility was measured by the Multidimensional Assessment of Interoceptive Awareness (MAIA). The findings show that Noticing, a subscale of the MAIA, significantly contributed to the worsening of psychological and somatic symptoms (all *p*s < 0.001). Conversely, Not-Distracting, Not-Worrying, Self-Regulation, and Trusting significantly contributed to the decrease of these symptoms (all *p*s < 0.05). The findings suggest that two aspects of interoceptive sensibility affected mental health in different ways during the mild lockdown. Mindfulness and mindfulness-based interventions would be effective in terms of enhancing adaptive aspects of interoceptive sensibility.

## 1. Introduction

The coronavirus disease 2019 (COVID-19) pandemic and quarantine have led to an increase in the number of people complaining of mental health problems. Depression, anxiety, and somatization symptoms increased at the outbreak’s peak in China [1]. Other adverse mental health conditions have also been pointed out, such as fear and insomnia [2,3]. Furthermore, some studies found that the impact of quarantine and isolation on mental health outcomes continued over a long period [4]. In Japan, “mild lockdown”, which was not enforceable and a non-punitive lockdown, was implemented [5]. Despite the loosely regulated lockdown, the number of railway users in the months under the mild lockdown decreased by 45.5% in April and 46.8% in May compared to each of the same months of the previous year [6,7]. Regarding the psychological impacts of non-coercive lockdown, Yamamoto et al. (2020) found that 36.6% of participants experienced mild to moderate psychological distress (Kessler Psychological Distress Scale (K6) score of 5–12) and 11.5% experienced severe psychological distress (K6 ≥ 13) [5]. Moreover, the estimated prevalence of depression (Patient Health Questionnaire-9 score ≥ 10) was 17.9% [5].

Many reports show an increase in mental health deterioration under quarantine, as mentioned above. Furthermore, partial lockdowns have been implemented in many countries due to the second wave of infections. Therefore, it is urgent to clarify the causes of the increase in mental health deterioration and factors that promote these symptoms and take measures to combat them during the quarantine.

Interoceptive sensibility is one factor associated with psychological disorders such as depression, anxiety, and somatization symptoms. Interoception is the perception of one’s body’s internal state such as the body-to-brain axis of sensation concerning the state of the internal body and its visceral organs [8]; the self-evaluated assessment of subjective interception is called interoceptive sensibility [8]. Interoceptive sensibility appears to be affected by the quarantine restrictions due to the current pandemic, and this decrease is related to deterioration in mental health. During past pandemics, enhanced sensibility to one’s bodily sensations was observed. For example, during the 2009 H1N1 influenza pandemic, many hospitals’ reported being flooded with “worried well” patients who mistakenly believed that their benign coughs of fevers were signs of pandemic influenza [9]. There is a concern about an increase in similar indefinite complaints in the current pandemic, and how to deal with them is an important issue [10,11]. It is assumed that this tendency of interoceptive sensibility is strengthened by the prevalence of unknown infection diseases, and long-term quarantine, making it easier to pay attention to one’s bodily sensations. Indeed, Lucia et al. (2020) measured the degrees of depression, anxiety, and interoceptive sensibility assessed by the Self-Awareness Questionnaire [12]. In a sample of amyotrophic lateral sclerosis (ALS) patients in March and April under the pandemic, it was found that all of the scales significantly increased in ALS patients compared to January and February before the pandemic [13]. The results suggest that interoceptive sensibility is enhanced during the lockdown and is related to mental health even under the current pandemic.

Most of the previous studies on interoceptive sensibility dealt with one aspect of interoceptive sensibility: paying attention to one’s bodily sensations is related to the onset of psychological disorders. However, recently, it has been argued that it is essential to understand interoceptive sensibility from multiple perspectives [14].

Interoceptive sensibility can be classified into two types depending on how people pay attention to interoception: the attention style based on anxiety and hypervigilance, and the mindfulness attention style [14]. The former view was common in the past, and paying attention to bodily sensations was regarded as maladaptive. Attention to somatic symptoms in clinical groups was regarded as representing anxiety, depression, and somatization symptoms [15]. Furthermore, in the study of anxiety and panic disorders, the terms “Body awareness” and “Somatic awareness” were used to describe cognitive attitudes characterized by an exaggerated focus on physical symptoms, somatosensory amplification, rumination, and catastrophic beliefs [16]. However, paying attention to bodily sensations is regarded as useful for health in the latter view [17]. Clinical research has pointed out that paying attention to bodily sensations such as mindfulness-based therapies in patients with various diseases leads to health benefits [18] and resilience-enhancing [14].

The Multidimensional Assessment of Interoceptive Awareness (MAIA) [17] was developed based on the background that there are two different interpretations of interoceptive sensibility, as mentioned above. The MAIA is a multidimensional self-report measure of interoceptive sensibility and consists of eight subscales: (1) Noticing, (2) Not-Distracting, (3) Not-Worrying, (4) Attention Regulation, (5) Emotional Awareness, (6) Self-Regulation, (7) Body-Listening, and (8) Trusting (Table 1). Although Noticing has been viewed as a subscale that does not distinguish between adaptive and maladaptive aspects [14], the subscale resembles previous scales developed based on the view that paying attention to bodily sensations exacerbates anxiety and hypochondriasis [14,19]. Thus, it can be considered to mainly measure the maladaptive aspect of interoceptive sensibility. Regarding the other subscales, it is said that they are about a mindful attention style [14], so they can be considered to measure adaptive aspects of interoceptive sensibility. Therefore, focusing on the adaptive and maladaptive aspects of interoceptive sensibility by using the MAIA can be used to comprehensively understand the effects of each element of interoceptive sensibility on people’s mental health.

Few studies have investigated the effects of interoceptive sensibility on mental health during the lockdown. In addition, it is not clear how each aspect of interoceptive sensibility is associated with psychological and somatic symptoms. Hence, in the present study, we aimed to examine how adaptive and maladaptive aspects of interoceptive sensibility affect depression, anxiety, and somatization symptoms under the mild lockdown related to the COVID-19 pandemic. We hypothesized that Noticing is associated with increased psychological and somatic symptoms. At the same time, the other seven subscales are associated with a decrease in these symptoms.

## 2. Materials and Methods

### 2.1. Participants

The survey was conducted online between 11 May and 12 May 2020 and was designed to assess the mild lockdown’s psychological impact on participants for approximately one month. Participants were recruited only in the seven prefectures where the non-coerced lockdown was implemented first. The number of people sampled in each prefecture was determined by the ratio of people living in each prefecture. 

Through Macromill, Inc. (Tokyo, Japan), approximately 80,000 people were recruited by email, and data were collected on an online platform. All participants voluntarily responded to the anonymous survey and provided informed consent online. These data are the same as those collected by Yamamoto et al. (2020) [5]. All data details were described more extensively by Yamamoto et al. (2020) and Sugaya et al. (2020) [20]. 

The exclusion criteria for participants were aged < 18 years; high school students; living outside the seven prefectures; and healthcare workers. Healthcare workers were excluded from analyses because they are generally considered to work more often than people who have other jobs. The online questionnaire created by Macromill, Inc. was designed in a way that none of the items could be skipped; that is, participants were not allowed to proceed to the next step if there were unanswered questions. Therefore, there were no missing data or study drop-outs in our survey. Finally, 10,672 participants were included in the analysis.

The Research Ethics Committee of the Graduate School of Social and Industrial Science and Technology, Tokushima University (no. 212) approved this study. It was performed according to the ethical standards of the 1964 Declaration of Helsinki and its amendments.

### 2.2. Measurements

#### 2.2.1. Demographic Data

Demographic information collected included age, sex, and a medical history. Regarding the medical history, information was collected on whether the individual was currently being treated for a psychiatric or physical illness, and whether the individual had a history of previous treatment for psychiatric or physical illness.

#### 2.2.2. Depression

Depression was measured using the Japanese version of the Patient Health Questionnaire-9 (PHQ-9) [21]. The PHQ-9 consists of nine questions, and participants reported depressive symptoms during the past four weeks, assessed by a score of 0 (not at all) to 3 (nearly every day) [22]. The PHQ-9 is widely used internationally as a screening scale for depression [23] with high reliability and validity [21]. In this study, highly reliability was observed (Cronbach’s α = 0.91).

#### 2.2.3. Anxiety

Anxiety was measured using the Japanese version of the Short Health Anxiety Inventory (SHAI) [24], which assessed hypochondriacal tendency (i.e., health anxiety). The SHAI accurately distinguishes between hypochondriacs and non-hypochondriacs [25] with high reliability and validity [25,26]. In this study, high reliability was also observed (Cronbach’s α = 0.84). Each item consists of four statements that are scored from 1 to 4 [26]. In this study, participants were asked to respond to two items (item number 5 and item number 12), which were selected in the order of descending factor loadings from among the 14 items of factor 1 (main section), which are considered to measure the main characteristics of health anxiety in general [24].

#### 2.2.4. Somatization Symptoms

Somatization symptoms were measured using the Japanese version of the Somatic Symptoms Scale-8 (SSS-8) [27]. The SSS-8 is an 8-item self-report questionnaire for somatic symptom burden. Participants reported somatic symptoms (e.g., back pain, headaches, and dizziness) during the past week, assessed by a score of 0 (not at all) to 4 (very much) [28]. The SSS-8 was developed as a shortened version of the Patient Health Questionnaire-15 (PHQ-15) [29], which is an internationally used scale to measure the presence and severity of somatic symptoms with high reliability and validity [27]. In this study, high reliability was also observed (Cronbach’s α = 0.82).

#### 2.2.5. Interoceptive Sensibility

Interoceptive sensibility was measured using the Japanese version of the Multidimensional Assessment of Interoceptive Awareness (MAIA) [30]. The MAIA is a 32-item self-report questionnaire with eight subscales, and items are answered from 0 (never) to 5 (always) [17]. This scale requires the scores for each subscale to be calculated, rather than the total score of all subscales [31]. The MAIA is highly reliable and valid [31], and in this study, high reliability was also observed (Noticing: Cronbach’s α = 0.82, Not-Distracting: Cronbach’s α = 0.85, Not-Worrying: Cronbach’s α = 0.75, Attention Regulation: Cronbach’s α = 0.95, Emotional Awareness: Cronbach’s α = 0.92, Self-Regulation: Cronbach’s α = 0.91, Body Listening: Cronbach’s α = 0.93, Trusting: Cronbach’s α = 0.90). Definitions of the eight scales are shown in Table 1.

### 2.3. Statistical Analyses

After calculating descriptive statistics of participants’ ages and each scale score, a correlation analysis was conducted to examine correlations between each MAIA subscale. The interpretation of the correlation coefficient was based on Mizumoto et al. (2008) [31].

Hierarchical multiple regression analysis was conducted for the PHQ-9, SHAI, and SSS-8 as objective variables. In order to control the effects of sex and age, we entered these variables in Step 1. To check for multicollinearity between the explanatory variables, the VIF was calculated. In general, it is said that we should pay attention to multicollinearity when VIF exceeds 10 [32]. As a result of the analysis, the VIF did not exceed 10 (all variance inflation factors < 5.09). We concluded that there was no risk of multicollinearity between any of the explanatory variables.

For all tests, significance was set at α = 0.05 (two-tailed). SPSS version 27.0 and RStudio version 3.6.0 were used for the analyses.

## 3. Results

### 3.1. Sample Characteristics and Correlation Analyses

A total of 11,333 people participated in this survey, and data from 10,672 participants (5191 males, mean age = 46.6 ± 14.6, age range = 18–89 years) who were not healthcare workers were used for analyses.

Demographic information and descriptive statistics of participants’ ages and each score are shown in Table 2. Additionally, the correlations coefficients between each subscale of the MAIA are shown in Table 3. As seen in the correlations between Not-Distracting and some subscales, there were negative correlations between the subscales (Attention Regulation: *r* = −0.23; Emotional Awareness: *r* = −0.29; Self-Regulation: *r* = −0.21; Body Listening: *r* = −0.21; Trusting: *r* = −0.11, all *p*s < 0.001), which are regarded as adaptive aspects.

### 3.2. Hierarchical Multiple Regression Analysis

Hierarchical multiple regression analysis was conducted for the PHQ-9, SHAI, and SSS-8 as objective variables.

Using the PHQ-9 as the objective variable, we entered the sex as a dummy variable and age in Step 1, and each subscale of the MAIA in Step 2. A summary of the results is shown in Table 4. The increment of the coefficient of determination is significant from Step 1 to Step 2 (Δ*R*^2^ = 0.21, *p* < 0.001). Additionally, as hypothesized, Noticing showed a significant positive effect (β = 0.13, *t* = 11.1, *p* < 0.001). Other hypotheses regarding other MAIA subscales were also largely supported and showed significant negative effects (Not-Distracting: β = −0.22, *t* = −19.9; Not-Worrying: β = −0.13, *t* = −12.9; Self-Regulation: β = −0.12, *t* = −6.43; Trusting: β = −0.26, *t* = −16.9, all *ps* < 0.001). However, in contrast to the hypotheses, significant positive effects were found for Emotional Awareness (β = 0.13, *t* = 7.21, *p* < 0.001) and Body Listening (β = 0.12, *t* = 7.11, *p* < 0.001).

As in the previous analysis, we conducted a hierarchical multiple regression analysis using SHAI as the objective variable. A summary of the results is shown in Table 5. The increment of the coefficient of determination is significant from Step 1 to Step 2 (Δ*R^2^* = 0.14, *p* < 0.001). Additionally, as hypothesized, Noticing showed a significant positive effect (β = 0.15, *t* = 11.9, *p* < 0.001). Other hypotheses regarding other MAIA subscales were also largely supported and showed significant negative effects (Not-Distracting: β = −0.07, *t* = −5.81, *p* < 0.001; Not-Worrying: β = −0.21, *t* = −19.7, *p* < 0.001; Attention Regulation: β = −0.04, *t* = −2.50, *p* < 0.05; Self-Regulation: β = −0.07, *t* = −3.32, *p* < 0.001; Trusting: β = −0.15, *t* = −9.26, *p* < 0.001). However, in contrast to the hypotheses, significant positive effects were found for Body Listening (β = 0.12, *t* = 6.55, *p* < 0.001).

We conducted a hierarchical multiple regression analysis using SSS-8 as the objective variable. A summary of the results is shown in Table 6. The increment of the coefficient of determination is significant from Step 1 to Step 2 (Δ*R^2^* = 0.29, *p* < 0.001). Additionally, as hypothesized, Noticing showed a significant positive effect (β = 0.20, *t* = 17.2, *p* < 0.001). Other hypotheses regarding other MAIA subscales were also largely supported and showed significant negative effects (Not-Distracting: β = −0.21, *t* = −19.1; Not-Worrying: β = −0.20, *t* = −20.5; Self-Regulation: β = −0.09, *t* = −5.14; Trusting: β = −0.20, *t* = −13.4, all *ps* < 0.001). However, in contrast to the hypotheses, significant positive effects were found for Emotional Awareness (β = 0.11, *t* = 6.56, *p* < 0.001) and Body Listening (β = 0.11, *t* = 6.33, *p* < 0.001).

## 4. Discussion

The purpose of this study was to examine how adaptive and maladaptive aspects of interoceptive sensibility affect depression, anxiety, and somatization symptoms under the mild lockdown related to the COVID-19 pandemic.

The hypotheses were generally supported: Noticing was significantly associated with increased maladaptive symptoms such as depression, anxiety, and somatization symptoms. Conversely, many of the other subscales of the MAIA were significantly associated with a decrease in these maladaptive symptoms. Regarding Noticing, the results of the present study are consistent with the view of previous studies. In earlier studies of interoceptive sensibility, Noticing was regarded to be the same as the aspect assessed by the questionnaires [33], and these questionnaires are related to mental disorders such as anxiety disorder (e.g., Steven Porge’s Body Perception Questionnaire [34]). In considering the consistency between our study’s results and those of previous studies, it is suggested that Noticing, which is considered to represent a maladaptive aspect of interoceptive sensibility, contributed to the increase of psychological and somatic symptoms even during the mild lockdown.

Conversely, Not-Distracting, Not-Worrying, Self-Regulation, and Trusting significantly contributed to the decrease in the PHQ-9, SHAI, and SSS-8. Moreover, Attention Regulation significantly contributed to the decrease in SHAI. As hypothesized, it was found that adaptive aspects of interoceptive sensibility had positive impacts on mental health even during the mild lockdown. Both de Jong et al. (2016) [35] and Bornemann et al. (2015) [33] proposed the effectiveness of mindfulness-based cognitive therapy and contemplative training for improving these adaptive aspects. These mindfulness-based interventions can be conducted at home, so they would be effective as training that we can work on during mild lockdowns.

However, some results differed from the hypotheses. Body Listening significantly contributed to the increase in the PHQ-9, SHAI, and SSS-8. Further, Emotional Awareness significantly contributed to the increase in the PHQ-9 and SSS-8. Both subscales have been regarded as adaptive aspects of interoceptive sensibility, but they negatively affected mental health in the present study. A possible reason behind these negative associations is that catastrophic thoughts about bodily sensations could be related. Both Body Listening and Emotional Awareness had significantly negative correlations with Not-Distracting (Body Listening: *r* = −0.21; Emotional Awareness: *r* = −0.29, all *p*s < 0.001). Therefore, these results indicate that higher Body Listening and higher Emotional Awareness is related to Distracting. Distracting from unpleasant bodily sensations is a common coping style that people with panic disorder [36] or chronic pain [14,35] often use. One reason this coping style is used is that people with panic disorder are susceptible to bodily sensations such as heartbeat. It is said that they often have catastrophic thoughts about these sensations and avoid these sensations because they see them as threatening [35]. Even in people with chronic pain, the Fear-Avoidance Model [37] explains the same things. This model most widely has been used to explain how chronic pain is sustained and deteriorates [38], and assumes catastrophic thoughts about bodily sensations and concomitant avoidance towards these bodily signals. A recent study also assumed these characteristics in this model [38]. Hence, the negative correlations of Body Listening and Emotional Awareness with Not-Distracting could imply that people exhibit catastrophic thoughts about bodily sensations. Although we pointed out the possibility that catastrophic thoughts existed as a background factor for the relationship between psychological or physical symptoms and Body Listening and Emotional Awareness, our research is not sufficient for determining this causal relationship. Therefore, it is necessary to investigate whether this relationship is observed by conducting a longitudinal study.

When future research determines the effects of catastrophic thoughts, interventions to mitigate them would be necessary. To alleviate catastrophic thinking toward bodily sensations, active exposures to interoception can be an effective intervention. Interoceptive exposures are applied to lessen panic attacks, which create a pseudo-oxygen-deprived state by exercise-induced increase in heart rate and breathing through straws [39,40]. These interventions aim to ease catastrophic thinking by repeatedly inducing and enduring bodily sensations and increasing coping potential [39,40]. Physical exercise is beneficial for physical and mental health and is recommended even during pandemic quarantine [41]. These interventions can be done quickly, even during quarantine, to be useful as a personal effort to alleviate catastrophic thoughts. 

The limitations of the present study are that although the survey was conducted during the mild lockdown, we did not take the exact number of days that individuals refrained from going out of their homes into account. Since the number of railway users under the mild lockdown decreased significantly, a large-scale tendency to refrain from going out was observed, but we did not examine the exact difference in individuals’ tendency to refrain from going out. Another limitation of the present study is that the causal relationship cannot be clarified because the survey was conducted at a single point in time. A follow-up study needs to be undertaken to examine this.

To the best of our knowledge, despite these limitations, this is the first study that focused on adaptive and maladaptive aspects of interoceptive sensibility and showed that each element had different mental health effects during the mild lockdown. Moreover, it was suggested that catastrophic thinking about bodily sensations might be increasing due to the current pandemic. The present study’s findings are essential as it highlights one of the secondary issues related to the current pandemic. 

## 5. Conclusions

In conclusion, Noticing significantly contributed to the increase of psychological and somatic symptoms. This suggests that a maladaptive aspect of interoceptive sensibility affects mental health deterioration even during the mild lockdown. In contrast to these results, Not-Distracting, Not-Worrying, Self-Regulation, and Trusting contributed to the decrease of these symptoms. This indicates that adaptive aspects of interoceptive sensibility have positive impacts on mental health. These results suggest that mindfulness and mindfulness-based interventions such as mindfulness-based cognitive therapy would be effective for enhancing these adaptive aspects of interoceptive sensibility during the mild lockdown. Body Listening and Emotional Awareness contributed to the increase of psychological and somatic symptoms, and we noted the possibility of catastrophic thoughts towards bodily sensations as background factors. However, future investigation through longitudinal studies is needed to confirm effects of catastrophic thoughts on interoceptive sensibility and mental health. 

## Figures and Tables

**Table 1:** Definitions of each subscale of the MAIA.

Scales	Definitions
Noticing	Awareness of uncomfortable, comfortable, and neutral body sensations
Not-Distracting	The tendency not to ignore or distract oneself from sensations of pain or discomfort
Not-Worrying	The tendency not to worry or experience emotional distress with sensations of pain or discomfort
Attention Regulation	Ability to sustain and control attention to body sensations
Emotional Awareness	Awareness of the connection between body sensations and emotional states
Self-Regulation	Ability to regulate psychological distress by attention to body sensations
Body Listening	Active listening to the body for insight
Trusting	Experience of one’s body as safe and trustworthy

**Table 2:** Sample characteristics.

	Yes	No	
Medical history			
Current treatment of psychiatric illness	592	10,080	
Previous treatment of psychiatric illness	1250	9422	
Current treatment of physical illness	448	10,224	
Previous treatment of physical illness	790	9882	
	***M***	***SD***	***Range***
Age	46.6	14.6	18–89
MAIA			
(1) Noticing	1.50	1.16	0–5
(2) Not-Distracting	3.58	1.21	0–5
(3) Not-Worrying	2.63	0.82	0–5
(4) Attention Regulation	2.16	1.23	0–5
(5) Emotional Awareness	2.17	1.23	0–5
(6) Self-Regulation	2.19	1.22	0–5
(7) Body Listening	1.97	1.26	0–5
(8) Trusting	2.26	1.25	0–5
PHQ-9	4.87	5.54	0–27
SHAI ^a^	1.23	1.42	0–6
SSS-8	6.61	5.59	0–32

^a^ We used the two items in the SHAI that are considered to reflect health anxiety best.

**Table 3:** Correlations between each MAIA subscale.

	(1) (95%CI)	(2) (95%CI)	(3) (95%CI)	(4) (95%CI)	(5) (95%CI)	(6) (95%CI)	(7) (95%CI)	(8) (95%CI)
**(1) Noticing**								
**(2) Not-Distracting**	−0.61 *** (−0.62–−0.60)							
**(3) Not-Worrying**	−0.43 *** (−0.45–−0.42)	0.49 *** (0.48–0.51)						
**(4) Attention Regulation**	0.37 *** (0.35–0.39)	−0.23 *** (−0.25–−0.22)	−0.04 *** (−0.06–−0.02)					
**(5) Emotional Awareness**	0.47 *** (0.46–0.49)	−0.29 *** (−0.30–−0.27)	−0.15 *** (−0.17–−0.13)	0.80 *** (0.79–0.81)				
**(6) Self-Regulation**	0.35 *** (0.34–0.37)	−0.21 *** (−0.23–−0.20)	−0.03 ** (−0.05–−0.01)	0.79 *** (0.78–0.80)	0.82 *** (0.81–0.82)			
**(7) Body Listening**	0.39 *** (0.37–0.40)	−0.21 *** (−0.23–−0.20)	−0.05 *** (−0.07–−0.03)	0.73 *** (0.72–0.74)	0.80 *** (0.79–0.80)	0.83 *** (0.83–0.84)		
**(8) Trusting**	0.27 *** (0.25–0.29)	−0.11 *** (−0.13–−0.09)	0.04 *** (0.02–0.06)	0.70 *** (0.69–0.71)	0.73 *** (0.72–0.74)	0.80 *** (0.79–0.80)	0.78 *** (0.78–0.79)	

*** *p* < 0.001, ** *p* < 0.01.

**Table 4:** Summary of hierarchical multiple regression analyses for variables predicting the PHQ-9.

Predictor Variables	Step 1				Step 2					
	*B*	*SE*	β	(95%CI)	*B*	*SE*	β	(95%CI)	*R* ^2^	Δ*R*^2^
Demographic variables									0.04 ***	
Sex	0.36	0.11	0.03 **	(0.01–0.05)	0.16	0.10	0.01	(−0.02–−0.03)		
Age	−0.07	0.00	−0.19 ***	(−0.21–−0.17)	−0.05	0.00	−0.14 ***	(−0.16–−0.13)		
Interoceptive sensibility									0.25 ***	0.21 ***
Noticing					0.62	0.06	0.13 ***	(0.11–0.15)		
Not-Distracting					−1.03	0.05	−0.22 ***	(−0.25–−0.20)		
Not-Worrying					−0.87	0.07	−0.13 ***	(−0.15–−0.11)		
Attention Regulation					−0.04	0.07	−0.01	(−0.04–0.02)		
Emotional Awareness					0.58	0.08	0.13 ***	(0.09–0.17)		
Self-Regulation					−0.55	0.09	−0.12 ***	(−0.16–−0.08)		
Body Listening					0.54	0.08	0.12 ***	(0.09–0.16)		
Trusting					−1.14	0.07	−0.26 ***	(−0.29–−0.23)		

*** *p* < 0.001, ** *p* < 0.01.

**Table 5:** Summary of hierarchical multiple regression analyses for variables predicting the SHAI.

Predictor Variables	Step 1				Step 2					
	*B*	*SE*	β	(95%CI)	*B*	*SE*	β	(95%CI)	*R^2^*	Δ*R*^2^
Demographic variables									0.00 ***	
Sex	0.07	0.03	0.03 **	(−0.01–0.05)	0.02	0.03	0.01	(−0.01–0.03)		
Age	0.00	0.00	−0.04 ***	(−0.05–−0.01)	0.00	0.00	0.01	(−0.01–0.03)		
Interoceptive sensibility									0.14 ***	0.14 ***
Noticing					0.18	0.02	0.15 ***	(0.13–0.17)		
Not-Distracting					−0.08	0.01	−0.07 ***	(−0.09–−0.05)		
Not-Worrying					−0.36	0.02	−0.21 ***	(−0.23–−0.19)		
Attention Regulation					−0.05	0.02	−0.04 *	(−0.07–−0.01)		
Emotional Awareness					0.04	0.02	0.03	(−0.01–0.07)		
Self-Regulation					−0.08	0.02	−0.07 ***	(−0.11–−0.03)		
Body Listening					0.14	0.02	0.12 ***	(0.08–0.16)		
Trusting					−0.17	0.02	−0.15 ***	(−0.18–−0.12)		

*** *p* < 0.001, ** *p* < 0.01, * *p* < 0.05.

**Table 6:** Summary of hierarchical multiple regression analyses for variables predicting the SSS-8.

Predictor Variables	Step 1				Step 2					
	*B*	*SE*	β	(95%CI)	*B*	*SE*	β	(95%CI)	*R^2^*	Δ*R*^2^
Demographic variables									0.01 ***	
Sex	0.77	0.11	0.07 **	(0.05–0.09)	0.45	0.10	0.04 ***	(0.02–0.06)		
Age	−0.02	0.00	−0.07 ***	(−0.09–0.05)	0.00	0.00	−0.01	(−0.03–0.00)		
Interoceptive sensibility									0.30 ***	0.29 ***
Noticing					0.95	0.06	0.20 ***	(0.17–0.22)		
Not-Distracting					−0.96	0.05	−0.21 ***	(−0.23–−0.19)		
Not-Worrying					−1.36	0.07	−0.20 ***	(−0.23–−0.18)		
Attention Regulation					0.03	0.07	0.01	(−0.02–0.04)		
Emotional Awareness					0.52	0.08	0.11 ***	(0.08–0.15)		
Self-Regulation					−0.43	0.08	−0.09 ***	(−0.13–−0.06)		
Body Listening					0.47	0.07	0.11 ***	(0.07–0.14)		
Trusting					−0.89	0.07	−0.20 ***	(−0.23–−0.17)		

*** *p* < 0.001, ** *p* < 0.01.

## Data Availability

The data presented in this study are available on request from the corresponding author.
